# Functional characterization of oligopeptide transporter 1 of dairy cows

**DOI:** 10.1186/s40104-017-0219-8

**Published:** 2018-01-23

**Authors:** Qingbiao Xu, Zhixuan Liu, Hongyun Liu, Fengqi Zhao, Xinbei Huang, Yueming Wu, Jianxin Liu

**Affiliations:** 1 Institute of Dairy Science, MOE Key Laboratory of Molecular Animal Nutrition, Zhejiang University, Hangzhou, 310058 People’s Republic of China; 2 College of Animal Sciences and Technology, Huazhong Agricultural University, Wuhan, 430070 People’s Republic of China; 3 Laboratory of Lactation and Metabolic Physiology, Department of Animal and Veterinary Sciences, University of Vermont, Burlington, VT 05405 USA

**Keywords:** Bovine, Kinetics, PepT1, Peptide absorption, Substrate specificity

## Abstract

**Background:**

It is well known that peptides play a vital role in the nutrition and health of dairy cows. Bovine oligopeptide transporter 1 (bPepT1) is involved in the peptide transport process in the gastrointestinal tracts of dairy cows. However, little information is known in the characteristics of bPepT1. Therefore, the purpose of this study was to characterize bPepT1 functionally using a mammalian cell expression system. The uptake of radiolabeled dipeptide glycyl-sarcosine ([^3^H]-Gly-Sar) into the bPepT1-transfected Chinese hamster ovary cells was measured at various pH and substrate concentrations and with or without 15 other small peptides that contained Met or Lys.

**Results:**

Western blot results showed that the abundance of bPepT1 protein in the jejunum and ileum are the highest in the gastrointestinal tract of dairy cows. The uptake of [^3^H]-Gly-Sar by bPepT1-Chinese hamster ovary cells was dependent on time, pH, and substrate concentration, with a low *K*_m_ value of 0.94 ± 0.06 mmol/L and a maximum velocity of 20.80 ± 1.74 nmol/(mg protein • 5 min). Most of the di- and tripeptides were the substrates of bPepT1, based on substrate-competitive studies. However, bPepT1 has a higher affinity to the peptides with shorter chains, greater hydrophobicity, and negative or neutral charges.

**Conclusions:**

These results demonstrated for the first time the functional characteristics of bPepT1, and they provide a new insight and better understanding into its vital role in absorbing a wide range of peptides from the digestive tract of dairy cows.

## Background

Peptide absorption from gastrointestinal lumen plays an important role in protein nutrition and health of animals [1, 2]. Peptides, which are hydrolyzed from dietary proteins, compose a considerable portion of soluble non-ammonia nitrogen in the gut [3] and the total amino acids (AA) in the portal-drained viscera of the ruminant [4]. Small peptides are taken up directly by the mammary gland for synthesis of milk proteins as a protein precursor [5, 6] and by many other tissues for nutritional and functional activities [2]. The oligopeptide transporter 1 (PepT1) plays a vital role in mediating the uptake of a fraction of dietary AA in the form of small peptides [1, 2]. Thousands of di- and tripeptides along with various peptide-like drugs, such as β-lactam antibiotics, angiotensin-converting enzyme inhibitors, and bestatin [7, 8], are absorbed in the apical membranes of enterocytes by PepT1 although they are taken up in the kidney, lung, mammary gland, liver, and brain by PepT2 [2, 9, 10].

PepT1 has been cloned and characterized in various species, including rabbit [11], human [12], sheep [13, 14], chickens [15], zebrafish [16], pig [17], turkey [18], cattle [19], Atlantic salmon [20], grass carp [21], and yak [22]. Most of the functional analysis of PepT1 has focused on the model peptides or pharmacological substrates, however, fewer reports have focused on the nutritional aspects of PepT1. Moreover, there are few reports on the PepT1 expression profile and kinetics in dairy cows.

The purpose of this study was to characterize the functional properties of bovine PepT1 (bPepT1) by measuring its transport kinetics and substrate specificities using radiolabeled model peptide [^3^H]-glycyl-sarcosine (Gly-Sar) in bPepT1-transfected Chinese hamster ovary (CHO) cells. This study provided new insights into better understanding of peptide absorption and better application of dietary peptides to accommodate bPepT1 function in the gastrointestinal tract of dairy cows.

## Methods

### Reagents

F12 Ham’s medium and fetal bovine serum (FBS) were purchased from Gibco (Grand Island, NY, USA). CHO cells were obtained from the Shanghai Bioleaf Biotech Co., Ltd. (Shanghai, China). [^3^H]-Gly-Sar (specific radioactivity: 1.85 GBq/mmol) was purchased from American Radiolabeled Chemicals (Saint Louis, MO, USA). Small peptides that contained *L*-type AA, except Gly which does not have *L*- or *D*-forms, were manufactured from China Peptides Co. (Shanghai, China). The primary rabbit anti-PepT1 (#ab78020; 1:1,000 dilution) and anti-β-actin antibodies (#ab8227; 1:5,000 dilution) were purchased from Abcam (Cambridge, UK), and the goat anti-rabbit secondary antibody was obtained from Beyotime (#A0208; Shanghai, China; 1:1,000 dilution).

### Cell culture and transfection

The CHO cells were cultured in F12 Ham’s medium that contained 10% FBS. All cell cultures were maintained at 37 °C and 5% CO_2_ in a humidified atmosphere. The culture medium was changed every other day. One day before transfection, the CHO cells were passaged and plated onto 12-well plates at a density of 5.0 × 10^4^ cells/well. The bPepT1 cDNA [19] was cloned into the pcDNA3.1 expression vector (Invitrogen, Carlsbad, USA). When the CHO cells were cultured to approximately 70–90% confluence, the cells were transfected with the pcDNA3.1-bPepT1 vector or the pcDNA3.1 vector as a control. For each well, 1 μg of DNA was mixed with 2 μL of P3000™ and 1 μL of Lipofectamine 3000 (Invitrogen, Carlsbad, USA) in 50 μL of OPTI-MEM and incubated at room temperature for 5 min. Then, the DNA-lipid complex was added into each well and the cells were transfected for 24 h.

### Western blot analysis

The tissue distribution of the bPepT1 protein in the gastrointestinal tract of dairy cows was analyzed by Western blot analysis, as described previously [23]. Briefly, the epithelial tissues of rumen, omasum, duodenum, jejunum, and ileum that were isolated from seven healthy Holstein dairy cows in mid-lactation were lysed and centrifuged, and the protein concentrations were determined using a BCA Protein Assay Kit (Beyotime, Shanghai, China). Equal amounts of proteins were electrophoresed on polyacrylamide gels and electro-transferred to a polyvinylidene difluoride membrane. The membranes were incubated overnight at 4 °C with either a rabbit anti-PepT1 antibody or an anti-β-actin antibody, and then incubated with an HRP-conjugated secondary antibody for 1 h at room temperature. The band densities were detected using a chemiluminescence system (CLiNX Science, Shanghai, China) and quantified using Quantity One software (Bio-Rad, Hercules, CA, USA). β-actin was used as a loading control to normalize the protein bands. The band intensity of bPepT1 expressed in the rumen was set to 1 as a baseline to compare those of all other tissues.

### Transport assay

The transport activity of bPepT1 was determined using [^3^H]-Gly-Sar (3.7 × 10^4^ Bq/mL), a well-known hydrolysis-resistant peptide substrate [24], in transiently transfected CHO cells. The transfected cells were washed three times with uptake buffer at pH 6.0 which contained 140 mmol/L NaCl, 5.4 mmol/L KCl, 0.8 mmol/L MgSO_4_, 1.8 mmol/L CaCl_2_, 5 mmol/L glucose, and 25 mmol/L MES. Then, the cells were preincubated with the buffer for 30 min at 37 °C.

For the transport time study, the transfected CHO cells were incubated with 0.02 mmol/L of [^3^H]-Gly-Sar (3.7 × 10^4^ Bq/mL) for various times (1, 5, 10, 20, 40, and 60 min). For the pH dependency study, the pH of the uptake buffer was adjusted to various pH (5.0, 5.5, 6.0, 6.5, 7.0, and 7.5) with HEPES/Tris or MES/Tris. For the transport kinetic study, Gly-Sar was prepared at various concentrations (0.02–10 mmol/L) with 3.7 × 10^4^ Bq/mL [^3^H]-Gly-Sar. For the inhibition study, 15 small peptides that contained essential AA at five concentrations (0.001–10 mmol/L) were added into the uptake buffer with [^3^H]-Gly-Sar, and they were incubated with the CHO cells for 5 min. Then, the concentration of unlabeled peptide that inhibited 50% of [^3^H]-Gly-Sar uptake (IC_50_) was calculated using PRISM software (GraphPad, San Diego, CA, USA). Uptake was stopped by washing with ice-cold uptake buffer. The cells were lysed by adding 0.5 mL lysis buffer that contained 1% Triton X-100, followed by incubation at 37 °C for 60 min. The radioactivity of the cell lysate was measured using liquid scintillation counting (LSC, Wallac1414, Wallac Co., Turku, Finland). The total protein of the cell lysate was determined using a BCA Protein Assay Kit (Beyotime). The uptake was expressed as nmol/(mg protein • 5 min).

### Sequence analysis

The membrane-spanning regions and orientation of bPepT1 were predicted using biological sequence analysis (Lyngby TMHMM Server; http://www.cbs.dtu.dk/services/TMHMM/). The AA sequences for potential N-glycosylation, cAMP-dependent protein kinase (PKA), and protein kinase C (PKC) sites were predicted using PROSITE computational tools (ExPASy Proteomics Server; http://prosite.expasy.org/).

### Statistical analysis

The values were presented as the means ± SEM. The data were analyzed using one-way ANOVA and Duncan’s multiple range tests using SAS software (SAS 9.2; SAS Institute Inc., Cary, NC). *P* < 0.05 was considered to be significantly different. Kinetic parameters and other calculations (linear as well as non-linear regression analysis) were determined using PRISM software.

## Results

### Structural features of bPepT1

**Fig. 1:**
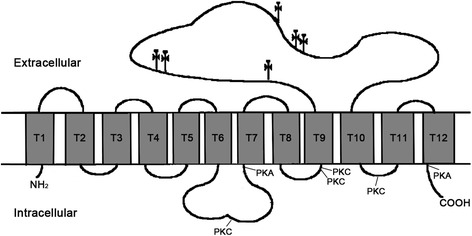
Putative membrane-spanning model of bovine peptide transporter (bPepT1). bPepT1 has 12 putative transmembrane domains with a large extracellular loop between transmembrane domains 9 and 10. The potential N-linked glycosylation sites are indicated by the symbol (†). The potential phosphorylation sites of protein kinase C (PKC) and protein kinase A (PKA) are also indicated

Sequence analysis showed that bPepT1 cDNA had an open reading frame of 2,121 bp that encodes a protein of 707 AA residues with an estimated molecular size of 78.4 kDa and an isoelectric point (pI) of 7.86. The bPepT1 AA sequence was highly conserved with PepT1 of goats (96.2% identity), sheep (95.2%), horses (85.9%), pigs (85.6%), dogs (83.4%), humans (83.0%), mice (82.7%), rats (81.6%), monkeys (81–83%), rabbits (78.6%), poultry (chickens and turkeys; 64–65%), and fish (56–61%). The hydropathy analysis predicted that bPepT1 has 12 potential transmembrane domains (TMD), with a large extracellular loop between TMD 9 and 10 (Fig. 1). Six putative extracellular N-glycosylation sites (Asn^406^, Asn^434^, Asn^438^, Asn^498^, Asn^508^, and Asn^513^) between TMD 9 and 10, four PKC sites (Ser^252^, Ser^266^, Ser^357^, and Ser^611^), and two PKA sites (Ser^255^ and Thr^670^) were identified in the bPepT1 sequence.

### Tissue distribution of bPepT1 in the gastrointestinal tract of dairy cows

**Fig. 2:**
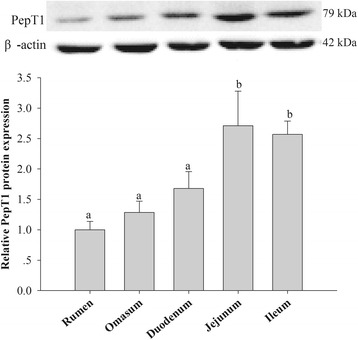
Tissue distribution of the bovine PepT1 protein in the gastrointestinal tract of dairy cows, as assessed by Western blot analysis. The values are means (*n* = 7) with their standard errors. Means without a common letter (top of the bars) are significantly different, *P* < 0.05

The bPepT1 protein was detected in the rumen, omasum, duodenum, jejunum, and ileum of dairy cows by Western blot analysis (Fig. 2). The levels of the bPepT1 protein in the jejunum and ileum were significantly higher than that in the rumen, omasum, and duodenum (*P* < 0.05), which suggested that the small intestine has a greater ability to absorb small peptides than the forestomach.

### Functional characterizations of bPepT1

**Fig. 3:**
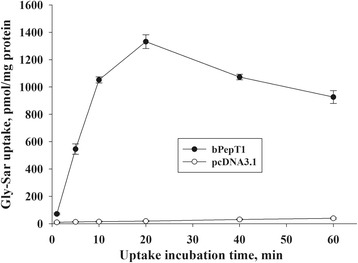
Time course of [^3^H]-Gly-Sar uptake in bPepT1-transfected Chinese hamster ovary cells. Gly-Sar uptake (0.02 mmol/L) in the transfected cells during a 1–60 min incubation. The values are means (*n* = 5) with their standard errors

**Fig. 4:**
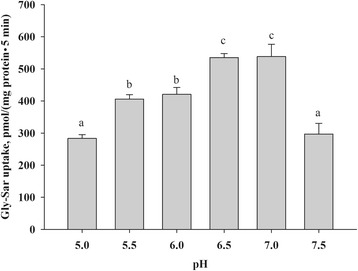
pH dependency of Gly-Sar uptake in bPepT1-transfected Chinese hamster ovary cells. The transfected cells were incubated with uptake buffer that contained 0.02 mmol/L [^3^H]-Gly-Sar (3.7 × 10^4^ Bq/mL) at pH 5.0–7.5 for 5 min. The values are means (*n* = 4) with their standard errors. The means without a common letter are significantly different, *P* < 0.05

After transfection with the bPepT1 expression vector for 24 h, the uptake of [^3^H]-Gly-Sar was measured (Fig. 3). The time course of [^3^H]-Gly-Sar uptake in transfected CHO cells showed that the uptake increased rapidly and linearly in the first 10 min and reached a plateau subsequently (Fig. 3). Therefore, in all subsequent experiments, an uptake time of 5 min was used. In contrast, the [^3^H]-Gly-Sar uptake in CHO cells transfected with the pcDNA3.1 control vector was only marginal, and it was much lower than in CHO cells that were transfected with the bPepT1 expression vector. The uptake in control cells increased slowly for 60 min (Fig. 3). In addition, in CHO cells that were transfected with the pcDNA3.1-bPepT1 vector, [^3^H]-Gly-Sar uptake was pH-dependent and greater at pH 6.5 and 7.0 than at pH 5.5 and 6.0 which was greater than at pH 5.0 and 7.5 (*P* < 0.05; Fig. 4).

**Fig. 5:**
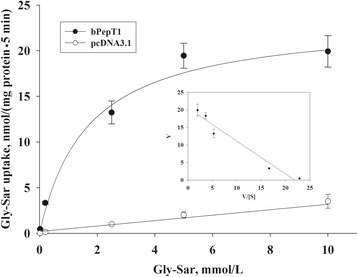
Kinetic analysis of Gly-Sar transport by bPepT1 that was measured in bPepT1-transfected Chinese hamster ovary cells. bPepT1-transfected (filled dots) and pcDNA3.1-transfected (empty dots) cells were incubated with five different concentrations of Gly-Sar (0.02–10 mmol/L, with 3.7 × 10^4^ Bq/mL [^3^H]-Gly-Sar) for 5 min. The cells transfected with the pcDNA3.1 control vector were used as a control. The values are means (*n* = 6) with their standard errors. *Inset*: Eadie-Hofstee plot of the Gly-Sar uptake in bPepT1-transfected cells after corrected by the uptake of empty vector-transfected cells at the individual concentrations

The Gly-Sar transport kinetics of bPepT1 was also measured in CHO cells that were transfected with bPepT1. As shown in Fig. 5, the transport rate increased with an increase in Gly-Sar concentrations, and it was saturated at 5 mmol/L. The transport data showed a good fit to the Michaelis–Menten equation. We estimated a *K*_m_ of 0.94 ± 0.06 mmol/L and a maximum velocity (*V*_max_) of 20.80 ± 1.74 nmol/(mg protein • 5 min) from the equation. The data were plotted as an Eadie-Hofstee graph (uptake rate/Gly-Sar concentration versus uptake rate) as a straight line (*r* = 0.93; Fig. 5, inset). In the subsequent experiments, a concentration of 0.02 mmol/L was used for bPepT1 substrate specificity.

**Fig. 6:**
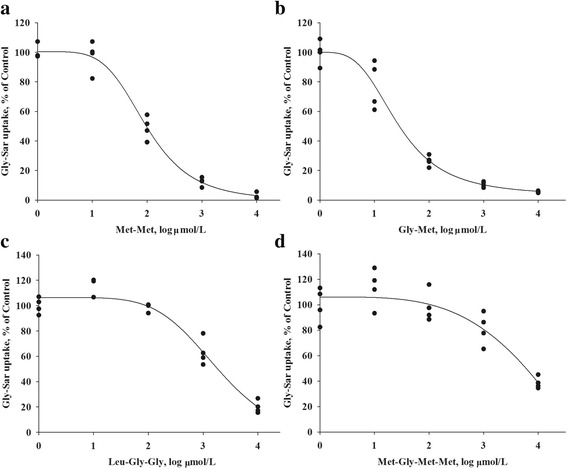
Transport of [^3^H]-Gly-Sar (0.02 mmol/L, 3.7 × 10^4^ Bq/mL) in bPepT1-transfected CHO cells in the presence of unlabeled di- (**a** and **b**, Met-Met and Gly-Met), tri- (**c**, Leu-Gly-Gly), and tetrapeptides (**d**, Met-Gly-Met-Met) at concentrations that ranged from 0.001 to 10 mmol/L for 5 min. Inhibition of Gly-Sar uptake was assayed at pH 6.0. The values are means (*n* = 4) with their standard errors

The [^3^H]-Gly-Sar uptake in the bPepT1 expression vector-transfected CHO cells could be inhibited by 15 other small peptides. Representative inhibition curves of 4 peptides (Gly-Met, Met-Met, Leu-Gly-Gly, and Met-Gly-Met-Met) were shown in Fig. 6. Ten dipeptides (Trp-Phe, Met-Lys, Met-Glu, Gly-Met, Lys-Met, Met-Gly, Gly-Lys, Met-Leu, Lys-Phe, and Met-Met) and one tripeptide (Met-Leu-Phe) had IC_50_ values that ranged from 0.014 to 0.096 mmol/L (Table 1). Four peptides (Lys-Lys, Leu-Gly-Gly, Lys-Trp-Lys, and Met-Gly-Met-Met) had IC_50_ values greater than 2.069 mmol/L. As expected, [^3^H]-Gly-Sar uptake was not inhibited in the presence of free AA Gly (data not shown). A comparison of kinetic parameters of bPepT1 from cows with PepT1 of sheep, pigs, and chickens was also shown in Table 1. In general, the substrate affinities of PepT1 from these four species were similar.

**Table 1:** Kinetic parameters of CHO cells transfected with bovine, sheep, pig, and chicken PepT1 cDNA

Substrate	Molecular weight	Charge^a^	Hydrop-hobicity^b^	pI	IC_50_, mmol/L			
					Bovine	Sheep^c^	Pig^d^	Chicken^e^
Gly-Lys	203	Positive	3.3	10.1	0.078	ND	ND	ND
Gly-Met	206	Neutral	1.5	5.5	0.032	ND	0.03	0.07
Lys-Lys	274	Positive	−7.8	10.0	9.649	0.739	3.8	7.9
Lys-Met	277	Positive	−2.0	8.8	0.032	0.051	0.04	0.11
Lys-Phe	293	Positive	−1.1	8.8	0.090	0.024	0.03	0.11
Met-Glu	278	Negative	−1.6	4.6	0.023	0.037	0.53	0.02
Met-Gly	206	Neutral	1.5	5.3	0.058	0.016	0.008	0.27
Met-Leu	262	Neutral	5.7	5.3	0.080	0.021	0.004	0.04
Met-Lys	277	Positive	−2.0	8.5	0.020	0.123	ND	0.07
Met-Met	280	Neutral	3.8	5.3	0.096	0.024	0.013	0.02
Trp-Phe	351	Neutral	1.9	5.5	0.014	ND	0.06	0.02
Leu-Gly-Gly	245	Neutral	3.0	5.5	2.069	0.127	0.27	0.08
Lys-Trp-Lys	460	Positive	−8.7	10.0	CBD	3.732	2.2	5.9
Met-Leu-Phe	409	Neutral	8.5	5.3	0.050	0.014	0.4	0.04
Met-Gly-Met-Met	468	Neutral	5.3	5.3	6.043	0.952	CBD	3.9

*pI* isoelectric point, calculated using p*K* values of the constituent amino acids, *IC*_*50*_ 50% inhibitory concentration, *ND* not determined; *CBD* cannot be determined

^a^ Charge was calculated at pH 6.0

^b^ Hydrophobicity was calculated as the average of the value of the constituent amino acids according to Kyte and Doolittle [38]

^c^ Sheep PepT1 data from Chen et al. [14]

^d^ Pig PepT1 data from Klang et al. [17]

^e^ Chicken PepT1 data from Chen et al. [15]

## Discussion

The bPepT1 protein has 12 putative TMD with a large extracellular loop located between TMD 9 and 10, consistent with the PepT1 structures of other species, such as rabbits [11], humans [12], rats [25], mice [26], sheep [13, 14], chickens [15], zebrafish [16], turkeys [18], and Atlantic salmon [20]. The bPepT1 protein has four potential PKC sites like the structure of sheep PepT1, but more than the PepT1 of other monogastric animals. The potential PKA sites were also found. These sites may be involved in regulation of the transport function of PepT1 by PKC and PKA [14, 27]. The predicted bPepT1 protein contains 707 AA, which is same in size to sheep and rabbit PepT1, but smaller than PepT1 from humans (708 AA), pigs (708 AA), rats (710 AA), mice (709 AA), chickens (714 AA), turkeys (714 AA), zebrafish (718 AA), and Atlantic salmon (734 AA). The similar functional characteristics between bPepT1 and ovine PepT1 should be resulted from the highest identity between the AA sequences of these two ruminants.

The expression of the bPepT1 protein was highest in the jejunum and ileum of the gastrointestinal tracts of dairy cows, which indicated that the small intestine may be the primary site of small peptide absorption; the small intestines of dairy cows had great potential to absorb peptides [28]. The pattern of PepT1 expression is consistent with previous studies [13, 29], in which the small intestine was the predominant site of *PepT1* mRNA expression in sheep and dairy cows. However, the observed expression of the bPepT1 protein in the rumen and omasum indicated that small peptides may be absorbed in the forestomach of dairy cows. This is in agreement with the results of our previous study that Gly-Sar could be absorbed readily in the primary omasal epithelial cells of dairy cows [30].

In this study, the functional activity of bPepT1 was characterized in a mammalian cell line, the CHO cell, which is a system used widely [14, 15, 17, 31, 32] to express mammalian genes similar to the *Xenopus oocyte* system [15]. In a previous study, the expression of ovine *PepT1* was observed after 8 h of transfection, and plateaued between 16 and 24 h [14]. Therefore, a time of 24 h after transfection was chosen for the study, when high expression and uptake activity of bPepT1 were validated. Given the large amount of small peptides that are hydrolyzed from dietary proteins and present in the gut for a short period of time, it seems reasonable that bPepT1 would transport peptides rapidly within minutes of time.

It is known that PepT1-mediated peptide transport is driven by an H^+^ gradient [10, 11], and it could be inhibited by low pH [33]. As predicted, Gly-Sar uptake by bPepT1 was pH-dependent, with a bell-shape curve observed in this study. In addition, the optimal pH (6.5–7.0) of bPepT1 may be relevant to the physiological pH of the forestomach (pH 6.2–6.8) and small intestine (pH 6.0–7.2) of dairy cows [34, 35], which would be beneficial to the absorption of small peptides in the physiological environment of the gastrointestinal tract. In this study, a pH of 6.0 was used in uptake assay, which aimed at to compare the data to PepT1 from sheep, chickens and pigs in previous studies. Moreover, the charge of the peptide was calculated at pH 6.0.

In addition, the calculated *K*_m_ value of bPepT1 for Gly-Sar was 0.94 ± 0.06 mmol/L in this study, which agreed with the low affinity of PepT1 in general, and this was similar to the *K*_m_ values from other species that included sheep (1.0 ± 0.1 mmol/L) [14], pigs (0.94 ± 0.14 mmol/L) [17], and chickens (2.6 ± 0.3 mmol/L) [15]. Furthermore, the *V*_max_ value of bPepT1 was 20.80 ± 1.74 nmol/(mg protein • 5 min) in this study, suggesting a possible greater capacity to transport peptides. These features of bPepT1 met the requirement for transporting a large number of various small peptides. The straight line of the Eadie-Hofstee graph indicated that a single transport system was responsible for Gly-Sar uptake in transfected CHO cells.

We also adapted the CHO cells expression system to study the competitive inhibition of bPepT1 transport by various short peptides. Most of the peptides used in this study consisted of essential AA, such as Met and Lys, which are considered the first limiting AA for dairy cows. The IC_50_ values varied with low values for 11 peptides, indicatingd higher bPepT1 binding affinity to these peptides. In particular, Trp-Phe was the most favorable peptide for bPepT1, immediately followed by Met-Lys and Met-Glu. High IC_50_ values were observed for four peptides (Lys-Lys, Leu-Gly-Gly, Lys-Trp-Lys, and Met-Gly-Met-Met), which indicated that they are less favorable substrates for bPepT1. The reason Lys-Lys and Lys-Trp-Lys were not the PepT1 substrates may be that they had two positively charged AAs [36]. In addition, the average IC_50_ value of tripeptides (4.36 mmol/L) was larger than that of dipeptides (0.18 mmol/L), consistent with that tripeptides generally have a much lower affinity for bPepT1 than dipeptides. A similar pattern was found in PepT1 of sheep, chickens, and pigs. It was reported that peptides longer than four residues and free AA are not PepT1 substrates [1, 2, 37]. Therefore, it can be concluded that the size of peptides plays a role in the transport process. In the 11 dipeptides tested, the average IC_50_ values of six dipeptides with neutral or negative charges (0.05 mmol/L) were smaller than the other five positive dipeptides (1.97 mmol/L), which indicated that positive charges may hinder transport. The reason may be that the positive charge of peptides could inhibit the binding of PepT1 to H^+^, which is required for peptide transport [36]. In addition, the affinity of bPepT1 for most peptides had values similar to PepT1 from sheep, chickens, and pigs. However, the affinity of bPepT1 for Leu-Gly-Gly was much lower than PepT1 from other species, perhaps, because a shorter uptake time (5 min) was used in this study, instead of the 20–40 min used in previous studies [14, 15, 17], which might have led to the hydrolysis of Leu-Gly-Gly. Moreover, bPepT1 had a higher affinity for peptides with higher hydrophobicity. These observations demonstrated that the PepT1 transport process could be influenced by various physical characteristics of the substrates, such as size, hydrophobicity, charge, and side chain flexibility. Furthermore, the interactions of these factors might also affect the transport activity of bPepT1 [36].

## Conclusions

In conclusion, a member of the proton-dependent oligopeptide transporter family, bPepT1, was characterized functionally for the first time. Western blot analysis demonstrated that the bPepT1 protein was expressed highest in the jejunum and ileum among the gastrointestinal tracts of dairy cows. Moreover, the bPepT1-mediated Gly-Sar uptake was time-, concentration-, and pH-dependent. In addition, the substrate-competitive studies of bPepT1 revealed that most of the di- and tripeptides were the substrates of bPepT1. Peptides with shorter chains, greater hydrophobicity, and negative or neutral charges could enhance bPepT1 activity. These results provide new insights into protein absorption in the digestive tract, which should lead to improved health and performance of dairy cows.

## Abbreviations

AA: Amino acid
bPepT1: Bovine PepT1
CBD: Cannot be determined
CHO: Chinese hamster ovary
FBS: Fetal bovine serum
Gly-Sar: Glycyl-sarcosine
IC_50_: 50% inhibitory concentration
ND: Not determined
PepT1: Oligopeptide transporter 1
pI: Isoelectric point
PKA: cAMP-dependent protein kinase
PKC: Protein kinase C
TMD: Transmembrane domains
*V*_max_: Maximum velocity
